# Scaling wood volume estimates from inventory plots to landscapes with airborne LiDAR in temperate deciduous forest

**DOI:** 10.1186/s13021-016-0048-7

**Published:** 2016-05-31

**Authors:** Shaun R. Levick, Dominik Hessenmöller, E-Detlef Schulze

**Affiliations:** 1 Department of Biogeochemical Processes, Max Planck Institute for Biogeochemistry, Hans-Knoell-Str. 10, 07745 Jena, Germany; 2 ThüringenForst AÖR, State Forest Service, P.O. Box 900105, 99104 Erfurt, Germany

**Keywords:** Broad-leafed, Carbon, Forest, LiDAR, Inventory, Wood volume, Temperate

## Abstract

**Background:**

Monitoring and managing carbon stocks in forested ecosystems requires accurate and repeatable quantification of the spatial distribution of wood volume at landscape to regional scales. Grid-based forest inventory networks have provided valuable records of forest structure and dynamics at individual plot scales, but in isolation they may not represent the carbon dynamics of heterogeneous landscapes encompassing diverse land-management strategies and site conditions. Airborne LiDAR has greatly enhanced forest structural characterisation and, in conjunction with field-based inventories, it provides avenues for monitoring carbon over broader spatial scales. Here we aim to enhance the integration of airborne LiDAR surveying with field-based inventories by exploring the effect of inventory plot size and number on the relationship between field-estimated and LiDAR-predicted wood volume in deciduous broad-leafed forest in central Germany.

**Results:**

Estimation of wood volume from airborne LiDAR was most robust (R^2^ = 0.92, RMSE = 50.57 m^3^ ha^−1^ ~14.13 Mg C ha^−1^) when trained and tested with 1 ha experimental plot data (n = 50). Predictions based on a more extensive (n = 1100) plot network with considerably smaller (0.05 ha) plots were inferior (R^2^ = 0.68, RMSE = 101.01 ~28.09 Mg C ha^−1^). Differences between the 1 and 0.05 ha volume models from LiDAR were negligible however at the scale of individual land-management units. Sample size permutation tests showed that increasing the number of inventory plots above 350 for the 0.05 ha plots returned no improvement in R^2^ and RMSE variability of the LiDAR-predicted wood volume model.

**Conclusions:**

Our results from this study confirm the utility of LiDAR for estimating wood volume in deciduous broad-leafed forest, but highlight the challenges associated with field plot size and number in establishing robust relationships between airborne LiDAR and field derived wood volume. We are moving into a forest management era where field-inventory and airborne LiDAR are inextricably linked, and we encourage field inventory campaigns to strive for increased plot size and give greater attention to precise stem geolocation for better integration with remote sensing strategies.

## Background

Temperate forests have functioned as significant sinks of atmospheric carbon dioxide CO_2_ over the last few decades, but their capacity for continued carbon sequestration is uncertain [1, 2]. Modelled estimates of the size and duration of the sink are highly variable, and reducing this uncertainty requires better quantification of how much carbon is stored in different forests types, how it is spatially distributed across environmental and land-management gradients, and how it changes over time. This task becomes increasingly urgent in view of the Paris Protocol where national sinks will balance national emissions. Estimates of above ground carbon stock have traditionally been measured and monitored through field-based inventories on a grid-scale [3]. These approaches typically rely upon allometric equations to scale simple field measures of tree structure (diameter at breast height, height) to wood volume—and ultimately to carbon mass by accounting for wood density [4, 5]. Allometric scaling has inherent limitations [6], but additional constraints of field-based inventories for regional scale analyses are the restricted spatial coverage of inventory plots, the time cost associated with conducting thorough wood volume estimations on the ground, and a lack of techniques to measure complete wood volume without the actual harvesting of stems.

Airborne light-detection and ranging (LiDAR) has emerged as a key remote sensing technology for advancing the mapping of forest structure and biomass over larger spatial scales [7–9]. The core strength of airborne LiDAR lies in its ability to accurately measure vegetation canopy height remotely, enabling detailed and georeferenced three-dimensional (3D) representations of canopy structure and associated biophysical parameters. Height and canopy density metrics derived from LiDAR have proven to be well correlated with above ground biomass (AGB) in a broad range of ecosystems—from semi-arid savannas to tropical forests [7, 10–13]. AGB mapping with airborne LiDAR is most commonly conducted by deriving empirical models between a suite of LiDAR metrics and field-measured AGB values obtained from georeferenced field sample plots. This relationship is then applied across the broader area of LiDAR data coverage at the same spatial resolution as the field sample plots from which the relationship was derived. As the number of studies comparing field-based estimates of AGB with LiDAR derived metrics has increased over the past decade, it has become increasingly apparent that performance is dependent upon the forest type and both the size and number of the field plots used for model development and evaluation [14–16]. Certain forest types lend themselves better to airborne characterization than others (e.g. conifers vs broad-leafed trees). Forests with relatively simple and regular structures, like those found in the boreal zone, are particularly well suited to characterization by airborne LiDAR [17–19]. Irrespective of forest type however, calibration errors between field measured and LiDAR predicted AGB tend to increase with decreasing plot size [15, 16]. This pattern partly arises from increasing edge effects as plots get smaller. Smaller plots have a greater edge length to area ratio than larger plots, and errors arising from GPS position uncertainty are also more pronounced in smaller than larger plots, as the same positioning offset will cause greater misalignment between field and LiDAR data in smaller than larger plots. Lastly, temporal differences between field and LiDAR data acquisitions can also strain the relationship between field and LiDAR measured AGB—due to natural growth/mortality, harvest, land-use and land-use change [20].

Despite the limitation of smaller plot size discussed above, sample plots of approximately 0.05 ha in size (25 m diameter) are standard for national forest inventories across much of the temperate zone [21]. As the science of forest management and forest inventory moves into a new era with greater inclusion of remotely sensed data to support monitoring and decision-making, we need better understanding of how well current field inventory approaches represent key forest variables at landscape to regional scales.

In this study we aimed to: (i) compare relationships between airborne LiDAR and wood volume estimates obtained from small (0.05 ha) and large (1 ha) field inventory plots; (ii) scale wood volume estimates from small (0.05 ha) and large (1 ha) inventory plots to the spatial extent of regional land-management units with airborne LiDAR; (iii) examine the consequence of using small (0.05 ha) or large (1 ha) field inventory plots for training airborne LiDAR extrapolations at the scale of land-management units; and (iv) determine the number of plots needed to adequately train LiDAR based extrapolations at landscape to regional scales.

## Methods

### Study site

This study was conducted in the Hainich-Dün region of Thuringia, Germany (51°12° N 10°18° E). Elevation ranges from 100 to 494 m above sea level and the region experiences a mean annual precipitation of 600–800 mm and a mean annual temperature of 6–7.5 °C. The parent material is limestone, which is covered in places by a loess layer of variable thickness (ca. 10–50 cm). Primary soil groups of the study area are Cambisols, Luvisols and Stagnosols [22]. The climate and soil conditions of the region provide optimum growing conditions for beech (*Fagus sylvatica* L.) dominated forests, with admixtures of *Fraxinus excelsior* L., *Acer pseudoplatanus* L. and *Acer platanoides* L.

At the turn of the 19th century, most of the forest sites in the Hainich-Dün region were under the coppice-with-standards system—a silvicultural system in which timber trees with an open canopy are grown above a coppiced woodland [23]. Small areas of selectively cut forests were also present, with selective harvesting of single trees and irregular forest use. In the early 19th century, all coppice-with-standards forests were converted to age-class forest or to selectively cut forest [24]. The forest under age-class management is characterised by a sequence of relatively homogenous, even-aged stands. Coppice-with-standards management is no longer practiced in the Hainich-Dün region.

### Field-inventory plot measurements

We used two different sets of existing field plots for comparison and extrapolation with airborne LiDAR data. The first set consisted of 50 large 1 ha plots (100 × 100 m) that were established as part of the Biodiversity Exploratories programme (see [25] for more details) to cover different forest and management types of deciduous forest within the region. The second set is a subset of the existing regional grid based forest inventory, totalling 1100 circular plots of 25 m diameter (0.05 ha) [26].

A comprehensive forest inventory was conducted in each of the 1 ha plots—with the species, height and stem diameter at breast height (DBH) recorded for all of trees with a DBH of >7 cm. In the 0.05 ha plots, a typical fixed area plot approach was used whereby: (i) all stems with DBH <=7 cm were measured within a 5 m radius of the plot centre point; (ii) stems with a DBH <12 cm were recorded within a 7 m of the plot centre point; (iii) stems with a DBH >12 cm were measured within a 12.5 m radius of the plot centre point. Thus, each inventory point yields information about stand density and diameter distribution when expanding to a common area. The conversion into wood volume follows allometric relationships, which include the form and taper of tree stems [5].

### Airborne LiDAR surveying

**Fig. 1:**
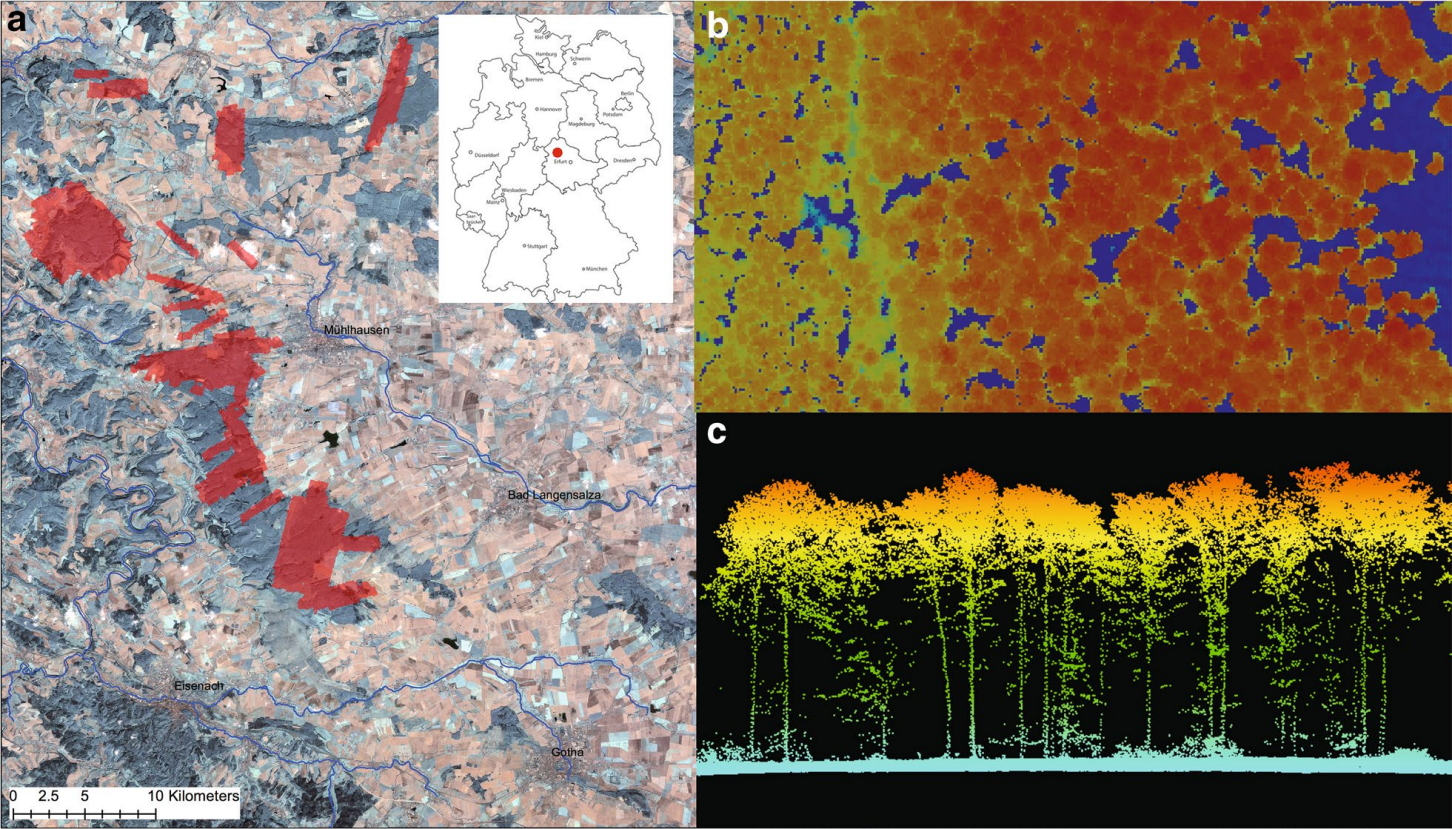
Aerial overview of study region in central Germany with LiDAR survey areas shown in *red* (**a**). Large overlap between flight lines and low flying altitude enabled high-resolution characterisation of forest canopy structure in both rasterised (**b**) and 3D point cloud (**c**) space

Airborne LiDAR surveying was conducted by Milan GmbH in August 2008 during leaf-on conditions. A Riegl LMS-Q560 full-wave form scanner (Riegl Laser Measurement Systems, GmbH, Horn, Austria) was operated at 240 kHz from 400–600 m above ground level. Beam divergence was 0.5 mrad and footprint size varied from 20–30 cm. An average pulse density of 16 per m^2^ and a mean point spacing of 0.24 m was achieved across the study site, providing excellent representation of the three-dimensional structure of canopy over 100 km^2^ of forest (Fig. 1).

### Airborne LiDAR processing

The geolocated LiDAR point clouds were projected into the UTM 32 N reference system and classified into ground and vegetation returns using the LAStools suite of processing tools (rapidlasso GmbH). A high-resolution digital terrain model (DTM) was interpolated from the ground-classified points at 1 m spatial resolution using a triangulated network (TIN) approach. The DTM was used to normalize the LiDAR point clouds to height above ground level. Field inventory plot centre locations were imported into the same projection system and buffered to create 25 m diameter circular polygons and 100 m wide square polygons centred on their respective inventory centre points. These polygons were used to clip and export the normalized LiDAR points falling inside each of the field inventory plots. The exported plot LiDAR points were then analysed in FUSION/LDV [27] to derive the suite of 25 structural metrics listed in Table 1, using a height threshold of 0.5 m above ground level to define vegetation returns.

**Table 1:** List of canopy structural metrics derived from airborne LiDAR

Canopy structural metric	Abbreviations
Total number of returns	totRET
Count of returns by return number	ret1, ret2, ret3, ret4, ret5, ret6, ret7, ret8, ret9
Minimum	minCH
Maximum	maxCH
Mean	MCH
Median	medCH
Mode	modCH
Standard deviation	stdev
Variance	var
Coefficient of variation	CV
Interquartile distance	intD
Skewness	skew
Kurtosis	kurt
Average absolute deviation	AAD
Median of the deviations from the overall median	MADmed
Median of the deviations from the overall mode	MADmod
L-moments (L1, L2, L3, L4)	L1, L2, L3, L4
L-moment skewness	Lskew
L-moment kurtosis	Kurt
Percentile values (5th–95th)	q1, q5, q10, q20, q25, q30, q40, q50, q60, q70, q75, q80, q90, q95, q99
Canopy relief ratio	CRR
Quadratic mean	CQM
Cubic mean	CCM
Canopy cover	cov
Canopy density	dens
Strata counts	s2, s4, s6, s8, s10, s12, s14, s16, s18, s20, s22, s24, s26, s28, s30, s32, s34, s36, s38, s40

### Establishing relationships between airborne LiDAR metrics and field-measured wood volume

We used two approaches to establish relationships between metrics derived from airborne LiDAR surveying and field-measured wood volume for both the 0.05 ha and 1 ha datasets. In the first approach we conducted step-wise linear regression with AIC minimisation to identify the LiDAR derived variables with the most explanatory power. In the second approach we employed machine learning using the Random Forest Algorithm on the same suite of variables and compared these results to those obtained from the simpler step-wise linear regression approach. In both cases we randomly selected 70 % of the data for training and used the remaining 30 % for cross validation.

### Exploring the consequence of using small or large field inventory plots for training airborne LiDAR extrapolations at the scale of land-management units

We used the best model (in terms of explanatory power and RMSE) for each plot size to extrapolate wood volume across the full extent of the available LiDAR coverage. We then intersected these regional wood volume maps with forest management GIS layers and compared the correlation between the extrapolated model derived from 0.05 ha plots and that derived from 1 ha plots on a land management unit basis.

### Establishing the influence of inventory plot sample size on wood volume relationships with airborne LiDAR

The number of inventory plots used in our study is considerably larger than most other studies linking airborne LiDAR to field estimates. In order to understand how increasing sample size influences the resulting relationship between field and airborne estimates, we developed a permutation simulation test in R to explore the effects of sample size on the correlation between the LiDAR metric with the most explanatory power and field-measured wood volume. Our approach involved: (i) the random selection of *x* plots from the full field dataset; (ii) fitting a linear regression between wood volume and LiDAR metric; (iii) repeating steps i and ii *y* times and quantifying the distribution of the regression outputs. For the 0.05 ha dataset, *x* ranged from 25–1000 in increments of 25 plots. For the 1 ha dataset, *x* ranged from 5–50 in increments of five plots. We ran 1000 permutations (*y*) for each sample size step in both datasets, resulting in a total of 40,000 linear regressions for the 0.05 ha dataset, and 10,000 linear regressions for the 1 ha dataset. We plotted box-plots of the R^2^ and the RMSE of from the linear regression outputs at each sample size step.

## Results

### Relationship between airborne LiDAR and field-measured wood volume

**Fig. 2:**
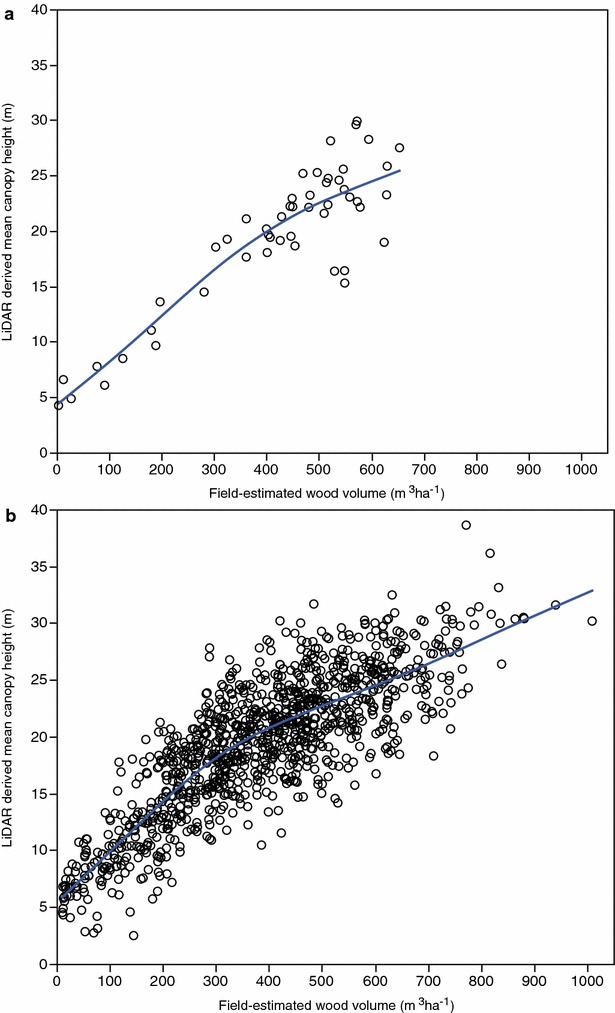
Relationship between field-estimated wood volume and LiDAR derived mean canopy height (MCH) at 1 ha (**a**) and 0.05 ha (**b**) *plot scales*

**Fig. 3:**
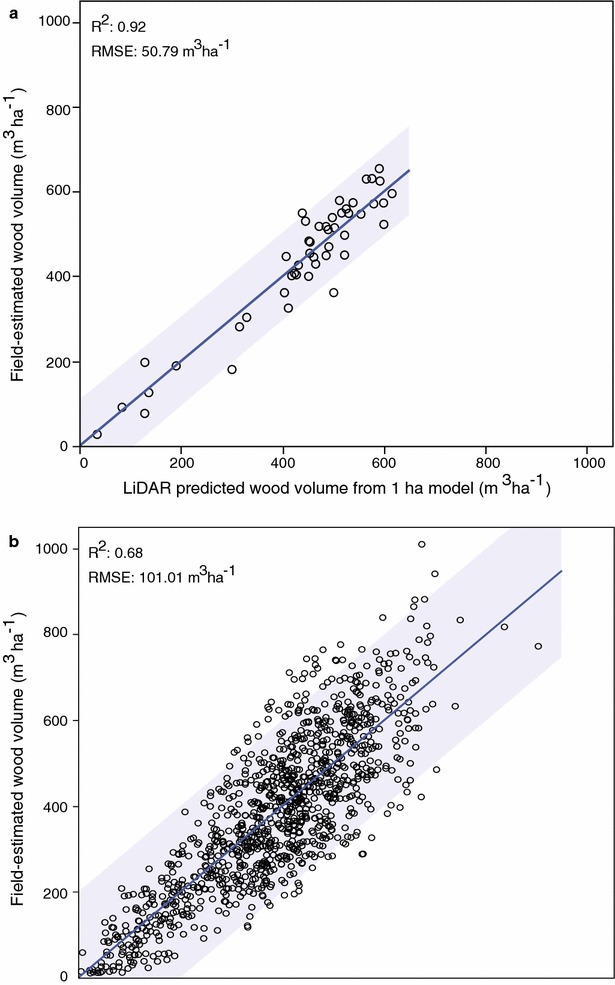
Validation of LiDAR-predicted wood volume against field-estimated wood volume at 1 ha (**a**) and 0.05 ha (**b**) *plot scales*

LiDAR derived mean canopy height (MCH) was well correlated with field-estimated wood volume at both the 1 ha and 0.05 ha scales (Fig. 2a, b). Step-wise multiple linear regression analysis showed that a combination of LiDAR derived height metrics could account for 92 % of the variation in field-measured wood volume at the 1 ha plot scale (R^2^ = 0.92, RMSE = 50.79, Fig. 3a). Five explanatory variables were retained in the final model (determined by AIC minimisation)—variance of canopy height (*var*), the 20th, 40th, and 70th percentiles (*q2, q4, q7*) and kurtosis (*kur*). The 70th percentile height (*q7*) was the most important explanatory variable, but inclusion of the other metrics reduced the RMSE.

$$\begin{aligned} Wood \,volume \,\left( {1 \,\rm{ha}} \right) = 120.32 + - 1.74\,(var) + - 4.38\,(q2) \quad \\ + 16.64\,(q4) + 9.7\,(q7) + - 11.05\,(kur) \end{aligned}$$

The same analysis for the 0.05 ha plots showed that LiDAR derived metrics could only account for 68 % of the variation in wood volume at this scale (R^2^ = 0.68, RMSE = 101.01, Fig. 3b). Mean canopy height was the most important explanatory variable, but CV was also significant and its inclusion reduced the RMSE.

$$\begin{aligned} Wood \,volume \,(0.05 \, {\rm{ha}}) =& - 144.85 + 25.89\,\left( {MCH} \right) \\ &+ \,67.64\,(CV) \end{aligned}$$

**Fig. 4:**
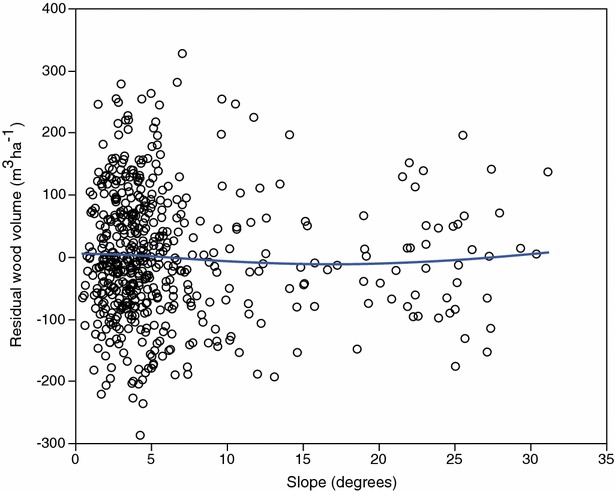
Pattern of LiDAR-predicted versus field-estimated wood volume model residuals (0.05 ha *plots*) in relation to terrain slope

There was a lot more scatter in the 0.05 ha relationships, and the RMSE was almost double that of the 1 ha scale plots. Evaluation of the model residuals showed no spatial pattern and there was no trend with increasing terrain slope (Fig. 4).

Random Forest modelling produced the same results and explanatory variables as the step-wise linear regressions, with only marginal improvements in RMSE. As such we used the simpler multiple linear regression equations for our landscape extrapolations.

### Extrapolating wood volume to landscape scale management units with airborne LiDAR

**Fig. 5:**
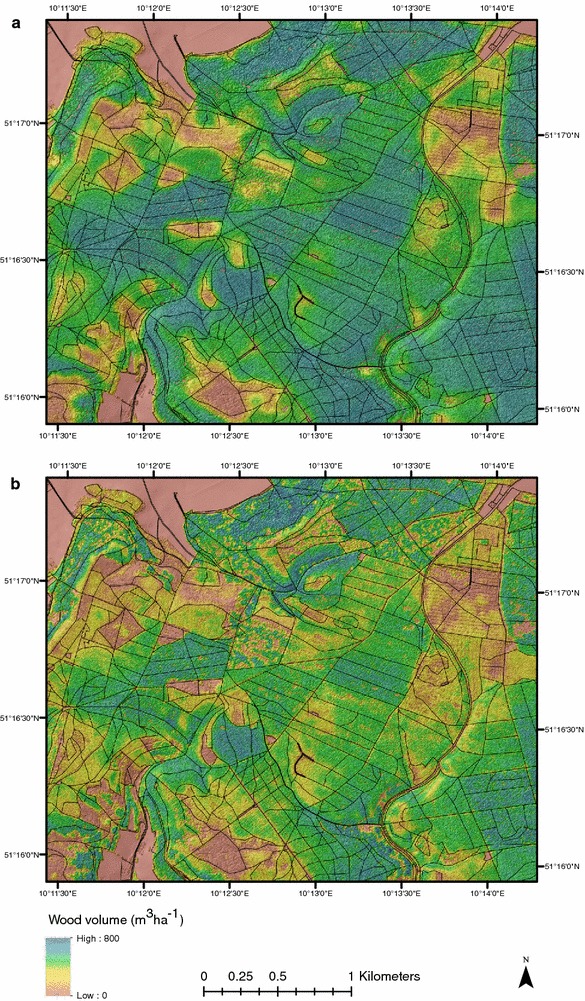
Landscape scale extrapolation of wood volume from the 1 ha model (**a**) and the 0.05 ha model (**b**). *Black lines* indicated forest management unit boundaries

**Fig. 6:**
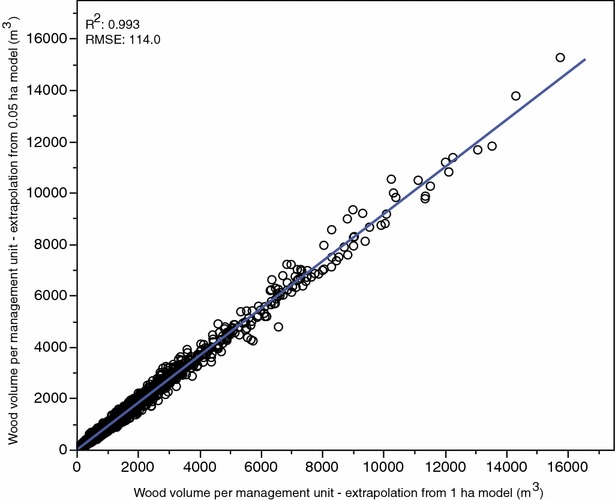
Relationship between total wood volume predictions from the 1 and 0.05 ha models on a per management unit basis

Extrapolation of wood volume from inventory plots to landscape scales with airborne LiDAR revealed a high degree of spatial variability in wood volume distribution, with the influence of forest management clearly evident in the patch characteristics (Fig. 5). The extrapolation from the 1 ha plots (Fig. 5a) produced a smoother distribution of wood volume with lower variance, as expected, whilst the 0.05 ha plot extrapolation retained higher spatial detail with greater variance (Fig. 5b). At the scale of individual forest management units, however, these differences are largely averaged out with strong linear correlation between LiDAR derived wood volume estimates from 1 and 0.05 ha models (Fig. 6).

### The influence of inventory plot sample size on wood volume relationships with airborne LiDAR

**Fig. 7:**
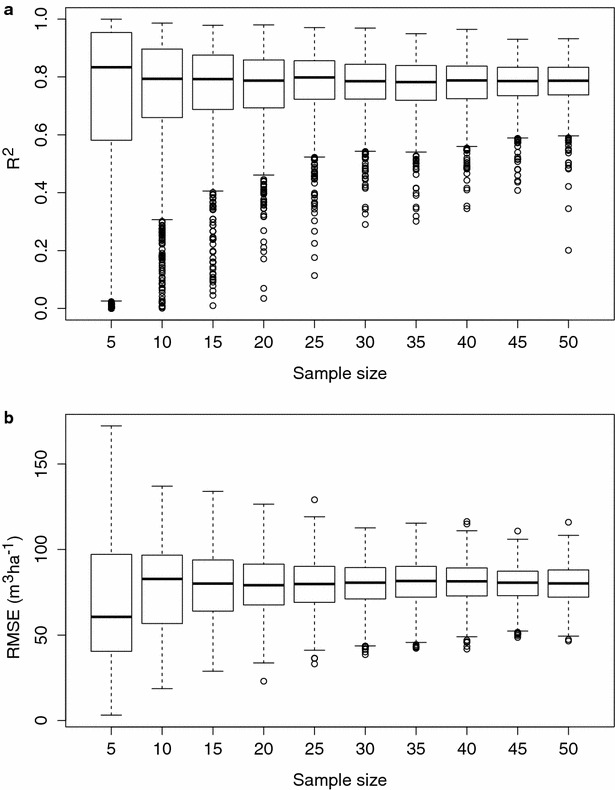
The influence of sample size (number of *plots*) on the proportion of variation in wood volume explained by LiDAR metrics—coefficient of determination (**a**) and root mean square error (**b**)—at the 1 ha *plot scale*

The median R^2^ value of the fit between field and LiDAR estimated wood volume at the 0.05 ha scale remained constant as the number of plots increased from 25 to 1000 (Fig. 7a). The variation around the median values decreased with increasing number of plots. With the smallest number of plots (n = 25) the range in R^2^ values spanned 0.37–0.91, and stability was only achieved with greater than 350 inventory plots. The same patterns held true for the RMSE, whereby the median values were consistent with increasing number of plots, but stability in the range between high and low values was achieved with plot numbers greater than 350 (Fig. 7b).

**Fig. 8:**
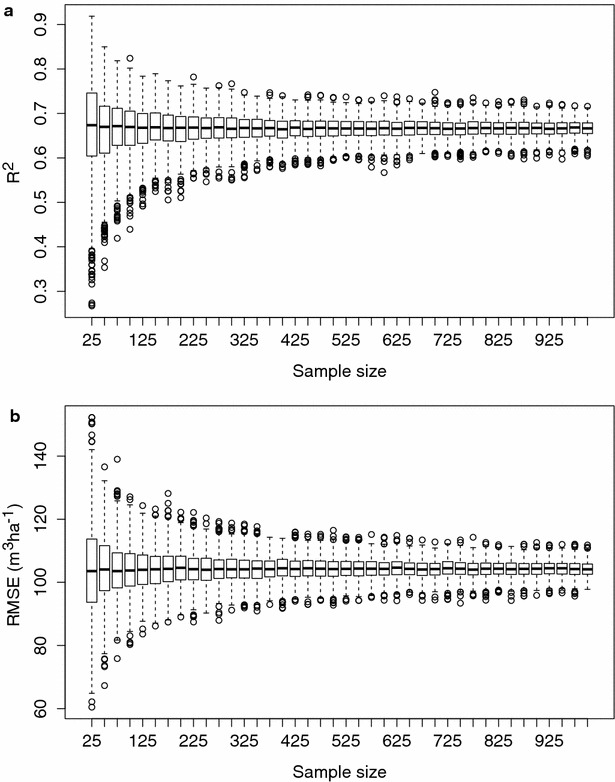
The influence of sample size (number of *plots*) on the proportion of variation in wood volume explained by LiDAR metrics—coefficient of determination (**a**) and root mean square error (**b**)—at the 0.05 ha *plot scale*

The pattern of decreasing range in R^2^ and RMSE values with increasing number of plots was repeated at the 1 ha scale (Fig. 8a, b). Median values where consistent at sample size of greater than 10 plots of 1 ha, and stability in the range between high and low values was achieved when the number of inventory plots was greater than 30.

## Discussion

Airborne LiDAR provides direct measurement of forest canopy height, but no information on DBH, which is the most commonly used (and often the sole) correlate of wood volume in field inventories. Establishing consistent and transferable relationships between wood volume and canopy structural variables that airborne LiDAR can acquire is important for enhancing forest inventory and long-term monitoring of aboveground biomass over large spatial areas. Our results from this study in central Germany confirm the utility of LiDAR for estimating wood volume in deciduous broad-leafed forest, but highlight the challenges of field plot size and number in establishing robust relationships between airborne LiDAR and field inventory derived wood volume.

Estimation of wood volume from airborne LiDAR was most robust (R^2^ = 0.92, RMSE = 50.79 m^3^ ha^−1^) when trained and tested with the 1 ha experimental plot data. Predictions based on the more extensive but considerably smaller (0.05 ha) inventory plots were inferior (R^2^ = 0.68, RMSE = 101.01 m^3^ ha^−1^). In above ground carbon terms, assuming a mean wood density of 0.57 g cm^−3^ [28] and a carbon content of 0.488 for temperate broad-leafed species [29], these findings relate to RMSE values of 14.13 Mg C ha^−1^ for the 1 ha plots and 28.09 Mg C ha^−1^ for the 0.05 ha plots. Higher error in the smaller plots was not unexpected, as larger plot sizes smooth out much of the variability inherent at smaller scales, e.g. the shelter wood harvest is only visible based on 0.05 ha resolution (Fig. 5). What was surprising however was the lack of any spatial pattern in the residuals of the fit between field-measured and LiDAR-predicted wood volume. We anticipated that plots situated on steeper slopes would deviate more from LiDAR-predicted values than those on flatter slopes due to variations in growth patterns, variations in canopy architecture and the increased difficulty of collecting geolocated field data in steep environments. This was not the case however as we found no spatial trends in the residuals and there was no significant relationships between slope attributes and the model residuals (Fig. 4). Furthermore, the even distribution of residuals above and below zero indicates equal probability of over- and under-estimation of wood volume from the 0.05 ha LiDAR model, suggesting a more random source of error.

Given the lack of environmental variation in the 0.05 ha model residuals and their uniform distribution, we consider edge effects to be the most likely cause of LiDAR prediction errors. Edge effects become more pronounced when plot size decreases, as greater proportions of tree canopy bisect the plot boundary [30]. During field inventory, trees rooted just inside the plot boundary contribute their full wood volume to the total plot estimate—yet any canopy extending over the boundary is ignored in the LiDAR analysis which clips the point cloud with the exact boundary dimensions of the field plot. This scenario would lead to an underestimation of plot wood volume from LiDAR. Similarly, any tree rooted just outside of a plot would not be recorded in the field wood volume inventory, but any of its branches and canopy that extend into the plot are included in the LiDAR analysis—leading to possible overestimations of plot wood volume from LiDAR. As such, these edge effects present equal opportunity for over- or under-estimations to arise, depending on the tree trunk geographic location in relation to the plot boundary line. These edge effect artefacts could be avoided, or at least minimised, by adopting a “crown-distributed” carbon density approach in the LiDAR analysis stage. Typical field inventories place carbon in space according to the geographic location of each stem (“stem-localised” approach), but as Mascaro et al. [15] and Packalen et al. [30] have shown, its makes more sense to distribute carbon spatially according to the foot-print of the tree’s crown (“crown-distributed” approach). The crown-distributed carbon density approach is more suitable for LiDAR-based investigations as LiDAR energy is returned more strongly by leaves and branches orientated perpendicular to the sensor, than stem boles orientated directly towards the sensor [15].

Successfully implementing a crown-distributed carbon density approach, however, relies on the delineation and identification of individual trees crowns in the airborne LiDAR data. Although much progress has been made in this direction through top-down segmentation of normalised canopy models [31–33], individual tree detection success is heavily dependant upon forest type. High accuracies have been recorded in coniferous forest and savanna, but success of individual crown delineation decreases as the complexity of canopy structure increases, and the interlocking crowns of broad-leafed temperate forest render them particularly challenging for automated individual crown separation [34–36]. Recent advances in bottom-up region growing techniques that identify trunk locations and segment their connected crowns within the LiDAR point cloud [35, 37] could prove useful in these forests. Airborne LiDAR datasets with higher pulse densities, preferably collected in leaf-off conditions, would be needed however to achieve sufficient returns from tree trunks to enable bottom-up delineation.

Despite the differences observed in the relationship between field-estimated and LiDAR-predicted wood volume at 1 and 0.05 ha plot scales, and the possible improvements that could be made in future LiDAR analyses, the greater uncertainty in the 0.05 ha model was of minimal consequence when scaling wood volume to land-management units across the entire landscape. We found hardly any difference between total wood volume estimates derived from the 1 and 0.05 ha models for individual land-management units (R^2^ = 0.99, RMSE = 114 m^3^). Nonetheless, reducing unexplained variation in the 0.05 ha model is important for ecological questions or management decisions operating at smaller scales, and for evaluating canopy dynamics over time. Although our results show minimal difference between 1 and 0.05 ha models at the scale of land-management units, we need greater exploration of how a broader range of plot sizes impact scaling relationships, across different forest types, to optimise the integration of field-based and airborne inventories.

In addition to advancing the LiDAR processing chain by adopting individual tree and crown-distributed carbon density approaches, uncertainty could be further reduced through improvements the field-inventory data collection. Common forest inventory systems in much of Europe utilise a fixed area sampling approach of three concentric circles of increasing distance from plot centre (5, 7, 12.5 m) and trees are sub-sampled according to DBH thresholds [38]. This approach assumes homogenous distribution of size classes across the plot, which is unlikely to hold true in reality. Measurement of all trees within the plot area would avoid this source of uncertainty, but involves greater time costs, and it would not significantly change the total volume estimate which depends on the coverage of the dominant trees. Nested fixed-area sampling approaches for forest inventory were developed to reduce sampling time per plot, and enable a higher number of plots to be sampled over more land area [39]. Our permutation tests in this study show however that very large plot numbers may yield limited benefit. Indeed, in terms of establishing relationships between field-estimated wood volume and airborne LiDAR metrics, increasing the number of plots beyond 350 does not improve the range of attained R^2^ and RMSE values (Fig. 6). In situations were field-inventory can be coupled with airborne LiDAR surveys, it would therefore make more sense to spend time and effort on increasing field plot size, and ensuring measurement of all stems, than increasing number alone. Moreover, the inclusion of airborne LiDAR into forest management and monitoring strategies can improve the effectiveness of field-based inventories by informing plot stratification over heterogeneous landscapes [12, 17, 40]. Lastly, inclusion of recent advances in terrestrial LiDAR sampling [41–43] into the field-inventory process would help reduce uncertainty of wood volume estimates at the plot scale, and facilitate integration of field and airborne data. Reducing uncertainty in the field to airborne scaling will be critical for validating upcoming global efforts to monitoring carbon stocks with spaceborne LiDAR, such as the global ecosystem dynamics investigation (GEDI) [44].

## Conclusions

The results from our study in broad-leafed deciduous forest show that airborne LiDAR can be used very effectively to map wood volume in deciduous forest stands, and therefore quantify carbon stocks across large landscapes at high spatial resolutions. We suggest that field inventory campaigns should prioritise plot size and place greater emphasis on precise stem and crown geolocation for better integration with high-resolution remote sensing techniques. Ensuring accurate geolocation of individual stems provides greater flexibility in the analysis stages of fusing LiDAR with field data, by enabling sub-sampling to provide information at a greater range of scales. We are moving into a forest management era where field-inventory and airborne LiDAR are inextricably linked. Forest inventory campaigns and airborne LiDAR surveying should not operate independently, as each add considerable value to the other.

## Abbreviations

DBH: diameter at breast height
LiDAR: light detection and ranging
RMSE: root mean square error
MCH: mean canopy height
